# The selective cytotoxic anti-cancer properties and proteomic analysis of *Trigonella Foenum-Graecum*

**DOI:** 10.1186/1472-6882-14-114

**Published:** 2014-03-29

**Authors:** Abdulaziz Alsemari, Fahad Alkhodairy, Ahmad Aldakan, Mai Al-Mohanna, Eman Bahoush, Zakia Shinwari, Ayodele Alaiya

**Affiliations:** 1 Department of Neurosciences, King Faisal Specialist Hospital and Research Centre, PO box 3354, Riyadh 11211, Saudi Arabia; 2 Department of Molecular Oncology, King Faisal Specialist Hospital and Research Centre, PO box 3354, Riyadh 11211, Saudi Arabia; 3 Proteomics Section, Stem Cell & Tissue Re-Engineering Program, King Faisal Specialist Hospital and Research Centre, PO box 3354, Riyadh 11211, Saudi Arabia

**Keywords:** Primary T-cell lymphoma, Cerebellum, T-lymphocytes cells, *Trigonella foenum graecum* (fenugreek), Brain tumor, Proteomics, Hierarchical cluster analysis/protein expression, Anti-cancer

## Abstract

**Background:**

There are a number of dietary components that may prove useful in the prevention and treatment of cancer. In some cultures, fenugreek seeds are used to treat cancer. The current study focuses on the anticancer properties and proteomic profiles of fenugreek seeds, and is prompted by the clinical profile of a case of primary CNS T cell lymphoma that responded to fenugreek treatment and resulted in tumor regression.

**Method:**

Various normal and cancer cell lines were exposed to fenugreek extract at differing concentrations (100 μg/ml, 200 μg/ml and 300 μg/ml) and at different time points (0, 24, 48, 72 and 96 hrs). Protein fingerprints of fenugreek grain/seed types, obtained from four different geographical regions, were analyzed by proteomic expression profiles.

**Results:**

We observed selective cytotoxic effects of fenugreek extract *in vitro* to a panel of cancer cell lines, including T-cell lymphoma. Additionally, the cluster analysis of proteomics data showed that the protein profile of the particular fenugreek used by the patient is significantly different from three other regional subtypes of fenugreek extract.

**Conclusion:**

The *in vitro* effect of fenugreek as a substance with significant cytotoxicity to cancer cells points to the potential usefulness of fenugreek in the prevention and treatment of cancer.

## Background

There is growing use of complementary and alternative anticancer medicines worldwide. *Trigonella foenum graecum* (fenugreek) is traditionally applied to treat disorders such as diabetes [1], high cholesterol [2], wound inflammation [3], and gastrointestinal ailments [4]. Fenugreek is also reported to have anticancer properties due to its beneficial active chemical constituents. It’s mechanism of action is similar to several anticancer drugs, and is based on an ability to induce apoptosis [5].

**Figure 1:**
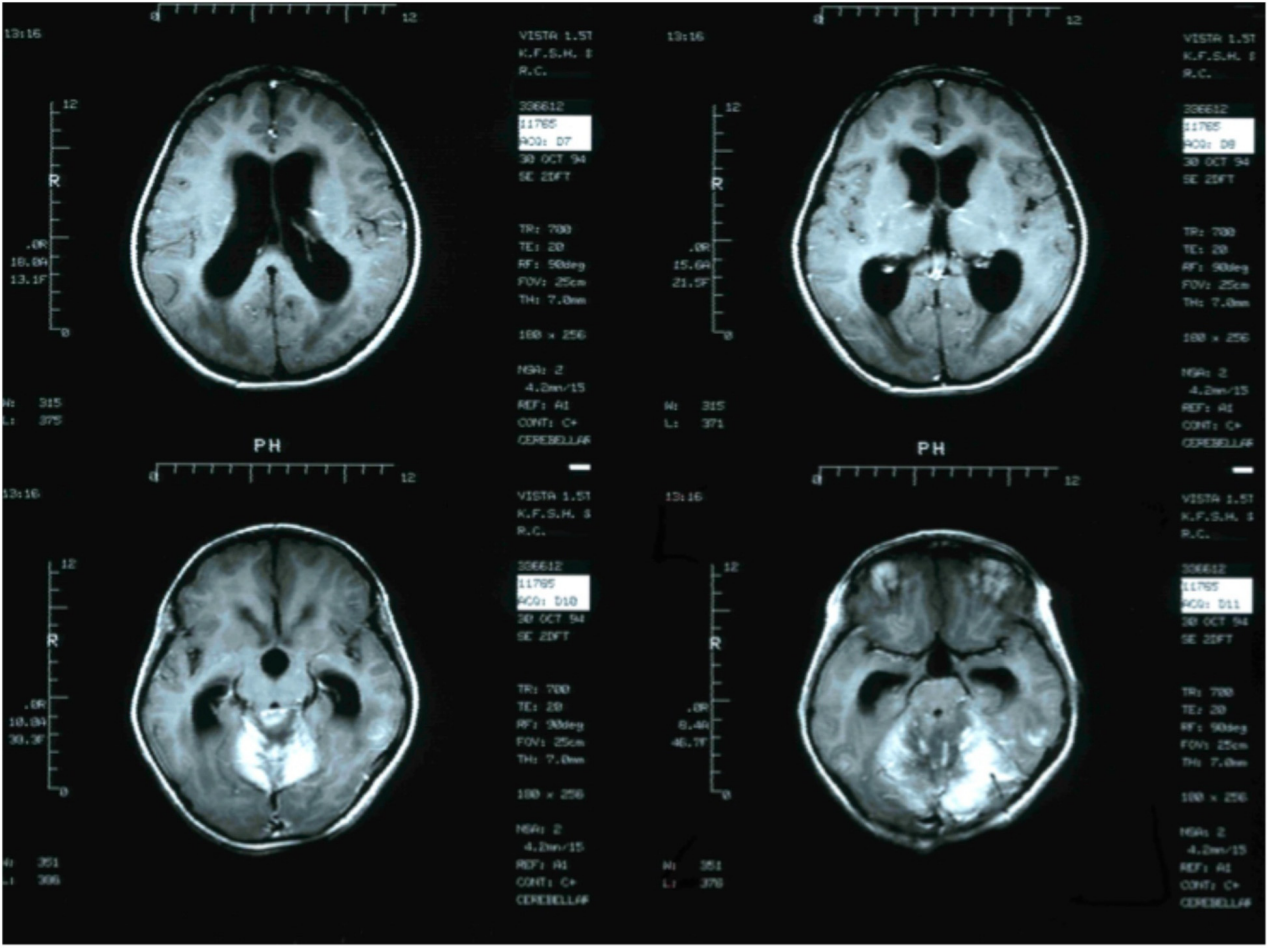
Brain MRI with contrast shows cerebellar enhancing lesions with secondary hydrocephalus.

The current *in vitro* studies were prompted by the clinical profile of a previously reported case of primary CNS T cell lymphoma [6]. Brain MRI with contrast was performed and revealed numerous cerebellar-enhancing lesions with secondary hydrocephalus (Figure 1). Brain biopsy of cerebral lesions showed a lympho-proliferative lesion. Histochemistry indicated T-cell infiltration of the cerebellum. Immunochemistry revealed immature T-lymphocytes (CD3, CD7, CD5) and mature lymphocytes (CD4, CD2).

**Figure 2:**
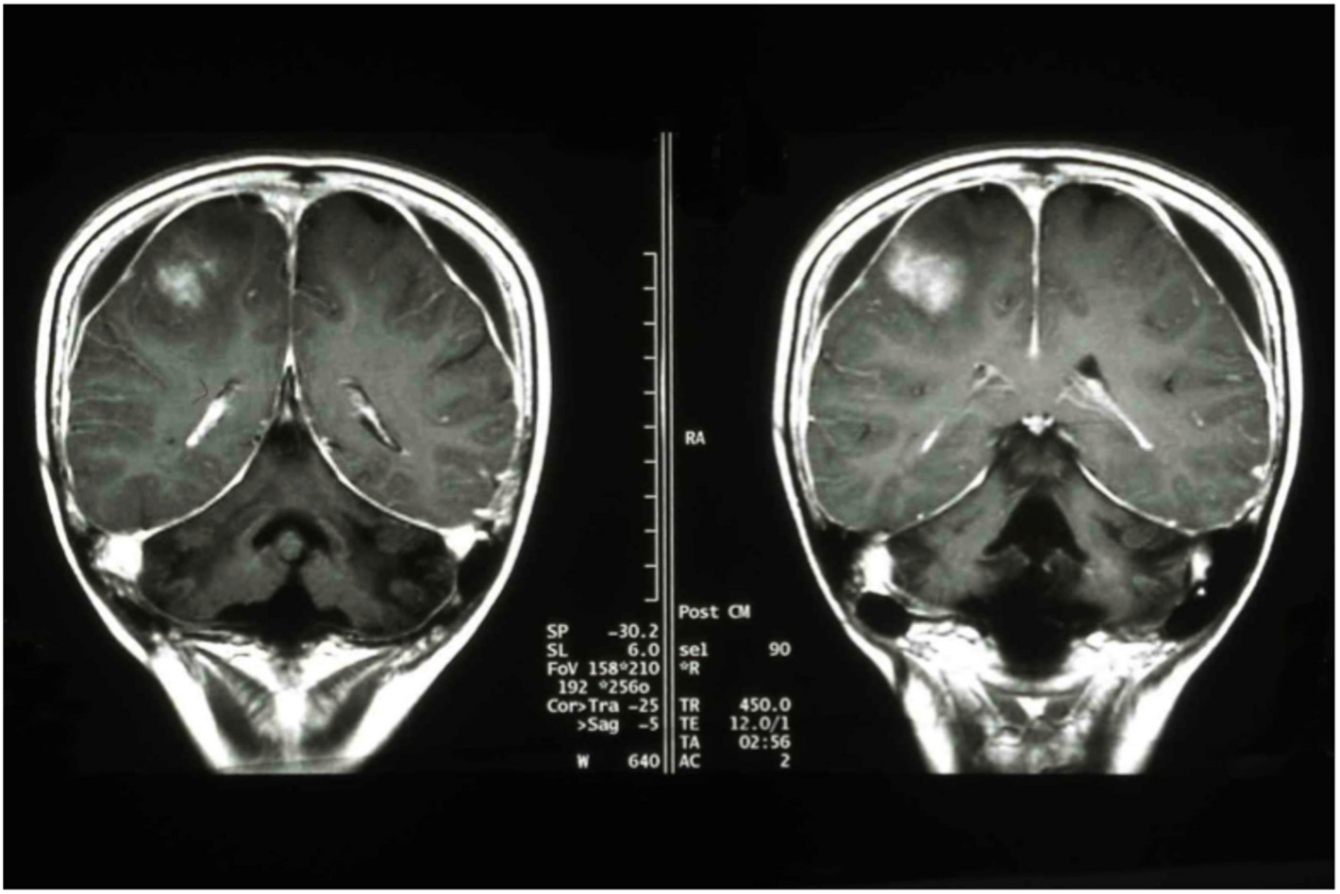
Brain MRI shows new enhanced lesion in the right frontoparietal lesion.

The patient initially went into clinical remission for 37 months after chemotherapy and radiotherapy. After three years of remission, the patient’s disease relapsed with intermittent weakness in her left arm and deviation of her mouth to the left. MRI of the brain with contrast showed a new enhanced lesion in the right frontoparietal region (Figure 2). The lesion was biopsied and the histopathology analysis confirmed the recurrence of her lymphoma. The patient was put on terminal care because the family refused treatment and took the child home [6].

**Figure 3:**
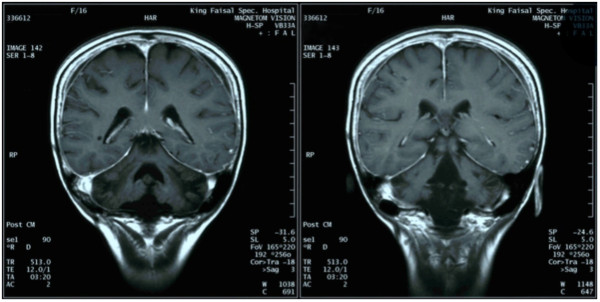
**Brain MRI with contrast shows *complete resolution of relapsed lymphoma.***

The family decided to treat their child with concentrated extract of fenugreek, boiled in regular water. The approximate daily treatment amount was 8 g of fenugreek seeds, over the course of 6 months. Repeat brain MRI three months later showed significant improvement in the new right intra cerebral lesion frontoparietal (Additional file 1: Figure S1). The subsequent brain MRI after six months showed complete resolution of the relapsed lesion (Figure 3). The patient continued in remission for 11 years; however, she had a devastating relapse in early 2012 and is currently emaciated and bed ridden. A repeat brain MRI in June 2012 showed new enhanced lesions and diffuse high signals on the right hemisphere (Additional file 2: Figure S2, ABCD).

In this report we demonstrate the selective cytotoxic effects of fenugreek extract *in vitro* on a panel of cancer cell lines, and a proteomics analysis of fenugreek.

## Methods

### Patient material

The patient consented for the publication of the clinical report. The study was approved by the Office of Research Affairs (ORA under the umbrella of Research Advisory Council (RAC) of King Faisal Specialist Hospital and Research Center (KFSHRC), with approval umber RAC#. 2120 006.

In order to avoid sample source variability all samples analyzed were purchased from the same local grocery store used by the patient.

### Cell culture and fenugreek extraction

A variety of normal and cancer cell lines including; T-cell lymphoma (TCP), B-cell lymphomas, Thyroid Papillary carcinoma (FRO) and human breast cancer (MCF7) were obtained from American Type Culture Collection (ATCC). Cells were cultivated at 37°C and 5% CO_2_ in Dulbecco’s MEM (DMEM) containing 5% fetal bovine serum, 3 mM glutamine (Gibco, NJ, USA) and antibiotics.

Fenugreek seeds were extracted in water, filtered, and concentrated with minor modification [7]. The extract was reconstituted to a working stock concentration of 50 mg/ml [8]. Cells were exposed to fenugreek extract at different concentrations (100 ug/ml, 200 ug/ml and 300 ug/ml) [9] and at different time points (0, 24, 48, 72 and 96 hrs).

### Cell viability assay

Cells were seeded onto a 96-well plate at a density of 5000 cells/well. In order to achieve meaningful quantitative analysis, duplicate or triplicates samples were subsequently incubated at 37°C for 72 hrs. in a titrated medium containing different concentrations of fenugreek extract. The number of viable cells was estimated using the protocol of 3-(4,5-dimethylthazol-2yl)-2,5-diphenyletrazlium bromide (MTT)-assay (Promega, Madison, WI, USA).

### Apoptosis analysis by annexin V staining

Approximately 5 × 10^5^ cells were seeded and cultured in 60 mm plates to 50-70% confluence. Triplicate samples were subsequently incubated at 37°C for 72 hours in a titrated medium containing different concentrations of fenugreek extract. The applied fenugreek extract was re-constituted in sterile water to yield the desired concentration of 300 ug/ml. Cells were collected at 24, 48, 72 and 96 hours, centrifuged and re-suspended in 1 ml of PBS. Thereafter, cells were stained with Propidium Iodide (PI) and Alexa Fluor 488 Annexin V, using Vybrant Apoptosis Assay kit #2 (molecular probes) according to the manufacturer’s protocol. Stained cells were analyzed by flow cytometry. Viable cell percentages were determined by the FACS Calibur apparatus and the Cell Quest Pro software from Becton Dickinson (BD Biosciences).

### Proteomics analysis of anticancer effects of fenugreek proteins on brain tumor

Protein patterns of different types of fenugreek grains/seeds were obtained from four different regions (labeled A-D). All four fenugreek types were analyzed by two-dimensional polyacrylamide gel electrophoresis (2-DE) for global differential protein expression profiles. The aim was to identify significantly differentially expressed proteins, and to identify proteins that are cancer related amongst the four types. The potential clinical applications of these proteins will be further evaluated.

### Electrophoresis, scanning and image analysis

Seeds/grains from four different fenugreek types were ground and protein extracted. The samples were diluted to a total volume of 350 μl, in a solution containing 8 M urea, 2 M thio urea, 0.2% Pharmalyte, 0.3% DTT, 2 M CHAPS and a trace of bromphenol blue. A total amount of 75 μg of protein was loaded on each strip via rehydration using linear pH 4–7 Ready IPG, strips (Bio-Rad, Hercules, CA, USA). First-dimension isoelectric focusing was carried out for a total of 45,500 Vh in a PROTEAN IEF cell (Bio-Rad).

The IPG-strips were then loaded and run on a 12.5% SDS-PAGE gel overnight at 100 V (constant) until the bromophenol blue dye front had reached the bottom of the gel. The gels were stained with silver nitrate and scanned using a calibrated densitometer, GS 800 and data was analyzed using PDQUEST software (Bio-Rad). Between 3–4 gels were run from each sample to allow for significant statistical analysis and ensure sample reproducibility.

### Data preprocessing/data analysis

A difference of ≥ 2-fold change was used as a threshold for marked quantitative difference between pairs of samples. Significantly differentially expressed protein spots were first selected using two different statistical methods (Student’s t test and Partial Least Square analysis), features available in PDQuest 2-DE image analysis software. The data from the match set was exported from PDQUEST in the form of a data table, with rows representing gels and columns representing spots. Datasets were normalized prior to analysis [10,11]. The resulting data were subjected to hierarchical clustering analysis using the J Express software (java.sun.com).

## Results

### Cytotoxic effect of fenugreek on normal and cancer cells

We found that fenugreek extract has a very selective cytotoxicity against cancer cell lines such as T-cell lymphoma (TCP), B-cell lymphomas, Thyroid Papillary carcinoma (FRO) and breast cancer (MCF7). On the other hand, there was no significant cell cytotoxicity amongst normal cells, including human lymphocytes and meningioma, when treated with fenugreek. This clearly indicates that fenugreek has selective cytotoxic effects against cancer cells (Additional file 3: Figure S3).

### Fenugreek selectively induces apoptosis in cancer and normal cells

**Figure 4:**
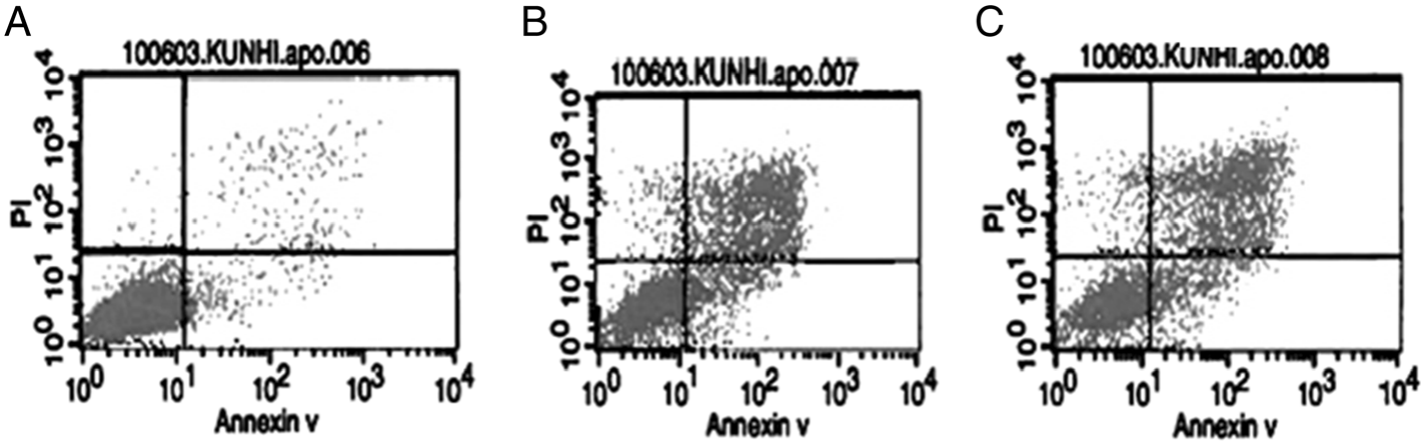
**Flow cytometric graphs of T-cell lymphoma without fenugreek treatment (A) and when treated with 300 μg/ml of Fenugreek, for 24, and 72 hours, B and C respectively.** The very low number of cells migrated to the right- upper panel are the dead cells by apoptosis induction (late apoptosis) the right lower is the early apoptosis indicating that the apoptotic cell cytotoxicity of fenugreek is very low in normal T-cell lymphocytes cell line. Apoptosis and necrosis was measured using Annexin V apoptosis assay kit. (Molecular Probe). The graphical distribution of viable cells, necrotic and population of apoptotic cells was gated according to their staining signals to PI and Annexin V. The data was generated using a FACS caliber flow cytometry.

Apoptosis and necrosis of normal and cancer cells was measured using Annexin V apoptosis assay kit Viable cells, necrotic cells, and apoptotic cells were gated utilizing PI and Annexin V staining. We observed significant fractions of apoptotic cells when normal T-cell lymphocytes and cancer cells (T-cell lymphoma-TCP, and human Thyroid papillary carcinoma-FRO) were treated with 300 μg/ml of fenugreek for 24 and 72 hours respectively. There was a high degree of both early apoptosis (Additional file 4: Figure S4 - cells in lower right corner) and late apoptosis (cells in upper right panel). The fractions of cells undergoing apoptosis due to fenugreek are significantly higher in all the studied cancer cells compared with normal cells (Figure 4, Additional files 4 and 5: Figure S4, S5 and Additional file 6: Table S1). All the data was generated using a FACSCaliber flow cytometer.

### Comparison of protein expression patterns between the four regional fenugreek samples

**Figure 5:**
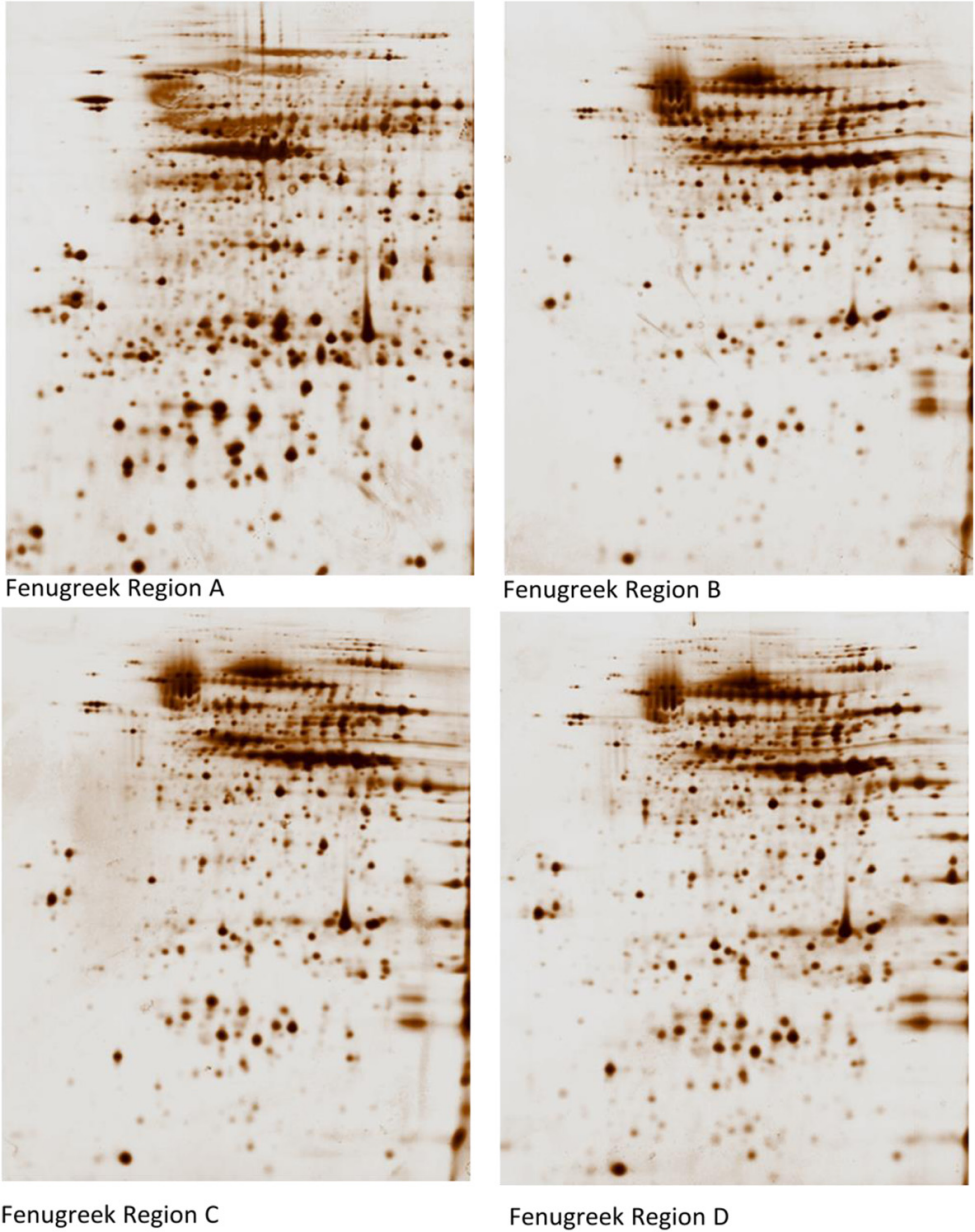
**Representative 2-DE gels derived from all the 4 different regional Fenugreek samples. (A)**: Fenugreek taken by the patient. **(B, C and D)**: Fenugreeks form other regions.

An average total number of 790 spots were resolved and minimum of 88.5% of the spots were matched between all the gels. Samples were run in triplicate gels to allow for analysis of statistical significance. Marked quantitative and qualitative similarities were observed in the protein expression profiles of all four fenugreek types. In addition, differential protein expression data revealed marked changes only in the expression profiles between sample A (The extract taken by the patient) and the other fenugreek types B, C & D. The similarity in protein expression between pairs of fenugreek samples was observed using the correlation analyses between two types of samples. When pairs of B/C/D samples were compared an average correlation coefficient of 0.86 was observed. However, we observed a significant degree of heterogeneity in the protein expression between pairs of A/B, A/C and A/D, with an average correlation of only 0.38 (Additional file 6: Table S2). Representative 2-DE maps from each of the fenugreek samples are shown in Figure 5.

### Hierarchical cluster analysis of differentially expressed proteins between the four regional fenugreek samples

**Figure 6:**
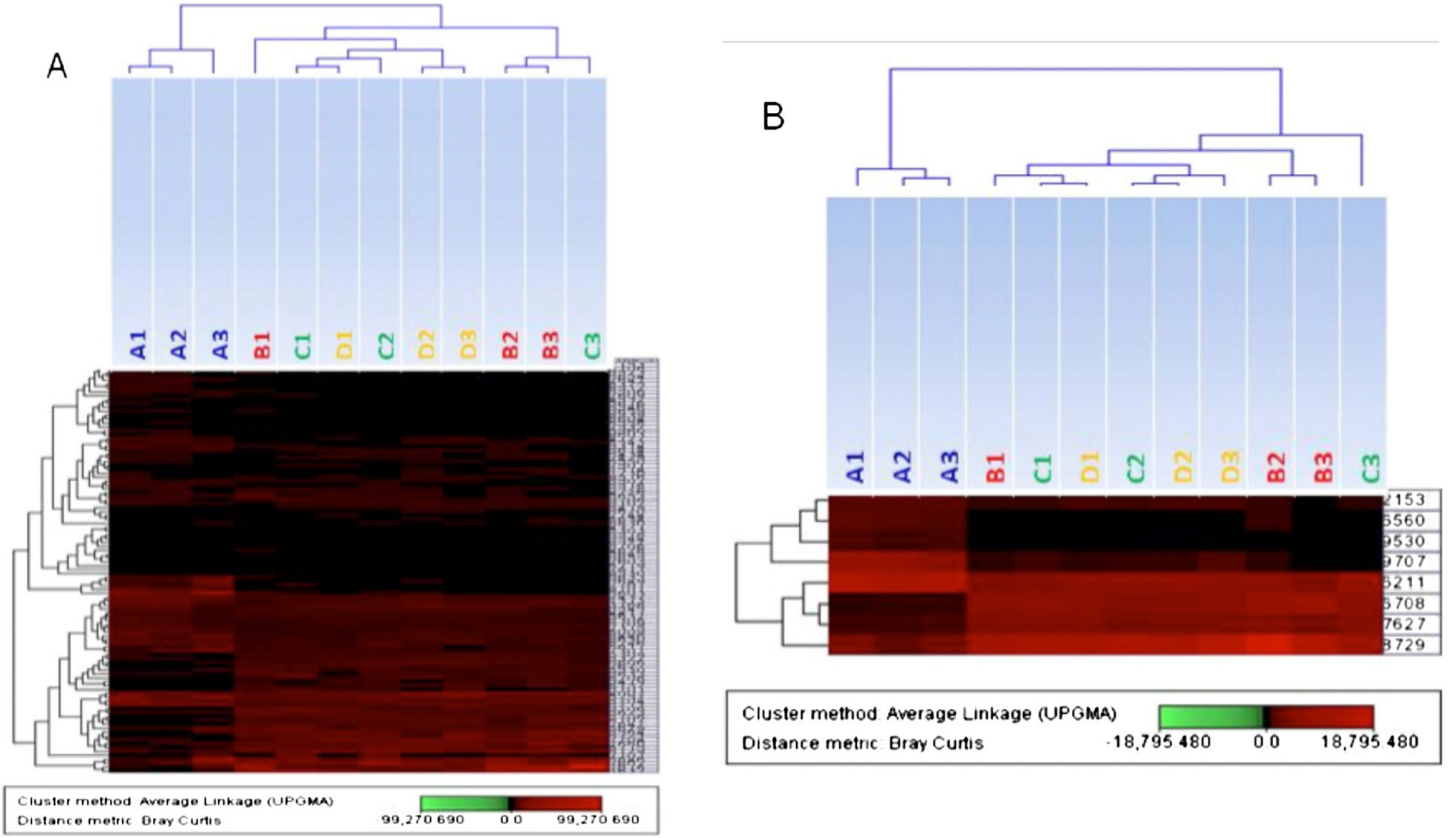
**Hierarchical Cluster analysis of fenugreek samples from region A away from the other three different regional fenugreek types using expression dataset from (A) 105 and (B) 8 protein spots that differed significantly using PLS and students-t test analyses respectively.**

Matched spots on the gels were compared to investigate differences in protein expression patterns. Protein expression data of a distinct set of 105 differentially expressed protein spots were examined. These differences were statistically significant using Partial Least Square analysis (PLS, P < 0.05) between sample A and the other three fenugreek types (B/C/D) combined. The resulting dataset from these 105 differentially expressed proteins were then subjected to hierarchical cluster analysis. A distinctive ‘heat map’ separating fenugreek A from the remaining three samples was observed (Figure 6A). Because of the limited sample size, we used another statistical method, Student’s- t test, to select variables that may discriminate between the three sample groups. This analysis resulted in 70 protein spots that differ significantly between the four sample groups. The resulting dataset from these 70 differentially expressed protein spots were then used for possible classification of the samples into their respective groups, using hierarchical cluster analysis. All the A samples were distinct from the other samples, similar to the pattern observed in Figure 6A (data not shown). To reduce the proteins within the dataset to a more practical number, a total of 8 protein spots, that fall in the intersection of the above two data sets, were selected. All fenugreek A samples were correctly classified away from the other three fenugreek sample types (B/C/D) using the 8 protein spot dataset (Figure 6B). The fact that the rest of the fenugreek types share more similarities in their expression patterns might explain why there is no distinct clustering of these three sample types. The localization on the 2-DE gel, as well as the differential expressions of some of these protein spots (shown as histograms), is indicated in Additional file 7: Figure S6.

Some of the differentially expressed protein spots, that are absent or down-regulated in the fenugreek A samples compared with the remaining three fenugreek types, are shown in Additional file 7: Figure S6. On the other hand, the up-regulated protein spots in the fenugreek A samples, as compared to the other fenugreek samples, are shown as gel segments in Additional file 8: Figure S7AB.

The clustering data, taken together with the correlation analyses of protein expression patterns, showed clearly that three fenugreek types (B/C/D) were very similar (average correlation coefficient = 0.86) compared with fenugreek A (r = 0.38). This indicates that even though there might be different fenugreek species, the medicinal properties they possess may be due to specific proteins that span the regional/species differences.

## Discussion

Despite the rarity of T-cell lymphomas, primary CNS lymphoma has a, distinctive clinical presentation [12-17], and has a poor prognosis [18]. The standard first-line treatment, of radiation therapy and chemotherapy, can achieve a complete response in only 20%–50% of patients. And the reported median survival for T-cell CNS lymphomas is between 13.5 and 22 months [17,19].

Our case study, to our knowledge, is the first of its kind to show the anticancer properties of fenugreek in a patient with established relapsed primary CNS lymphoma. The daily use of aqueous extract of fenugreek resulted in 11 years remission of relapsed primary CNS T- cell lymphoma. Also our experimental research, *in vitro,* showed that fenugreek is also selectively cytotoxic to cancer, and not to normal cells. Normal lymphocytes showed no apoptosis while T-Cell lymphoma and breast cancer cells were highly apoptotic following fenugreek treatment.

There is a clear reciprocal *in vivo* and *in vitro* anti cancer response to aqueous fenugreek. The distinctive, long duration, remission of a patient with relapsed primary T-cell lymphoma after daily use of aqueous extract of a particular *Trigonella foenum graecum*, however remarkable, only represents a single case. A more rigorous scientific evaluation, such as a clinical trial, is obviously warranted before the anti cancer properties of fenugreek can be confirmed

Nonetheless, alternative medicine use is widespread amongst cancer patients. In many surveys, herbal medicines are amongst the most commonly used treatments. Herbal remedies are believed by the general public to be safe, cause less side-effects and be less likely to cause dependency [20]. However most of these perceptions have not been studied in prospective research.

Extracts of fenugreek seeds, and some of their constituents, have been shown to have anti carcinogenic potency [21-23]. Anti cancer properties of fenugreek, or its constituents, involve multiple functional and molecular targets. Fenugreek induced apoptosis has been reported in a wide variety of tumor cell lines including human; colon [24], osteosarcoma [25], leukemia [26], breast [27], and liver [28].

The preventive efficacy of dietary fenugreek seed, and its major constituent, diosgenin, on azoxymethane-induced rat colon carcinogenesis during initiation and promotion stages has been evaluated [24]. These *in vitro* experiments indicated that diosgenin inhibits cell growth and induces apoptosis in the HT-29 human colon cancer cell line in a dose-dependent manner. Furthermore, diosgenin induced apoptosis in HT-29 cells at least in part by inhibition of bcl-2 and by induction of caspase-3 protein expression. This study suggests the fenugreek constituent, diosgenin, has potential as a novel preventive agent for colon cancer [24].

Another study suggested significant chemo preventive effect of fenugreek seeds against breast cancer [29]. Fenugreek seed extract was demonstrated to significantly inhibit MDA 231-induced mammary hyperplasia and decreased its incidence. The authors further suggest that apoptosis might mediate fenugreek’s protective anti-breast cancer effects [29]. The induction of apoptosis by fenugreek extract is suggested by its ability to increase the expression of pro-apoptotic genes, and thus this spice holds promise for consideration in complementary therapy for breast cancer [5].

In a recent study, fenugreek was demonstrated to be cytotoxic to a panel of cancer but not normal cells [23]. Treatment with 10–15 μg/mL of fenugreek for 72 h was found to be growth inhibitory to breast, pancreatic and prostate cancer cell lines. When tested at higher doses (15–20 μg/mL), fenugreek continued to be growth inhibitory to prostate cancer cell lines but not to either primary prostate or normal prostate cell lines. At least part of the growth inhibition was reported to be due to induction of cell death, as seen by incorporation of Ethidium Bromide III into cancer cells exposed to fenugreek [23].

Protodioscin (PD) has been purified from fenugreek and identified by Mass, and 1H- and ^13^C NMR. The effects of PD on cell viability in human leukemia (HL-60) and human stomach cancer (KATO III) cells have been investigated. PD displayed strong growth inhibitory effect against HL-60 cells, but weak growth inhibitory effect on KATO III cells. Morphological changes (apoptotic bodies) have been observed in HL-60 cells treated with PD, but not in KATO III cells treated with PD. These findings suggest that growth inhibition of HL-60 cells by PD results from the induction of apoptosis [21].

Al-daghri [30] reported that incubation of Jurkat T lymphocytes cells with fenugreek extract at concentrations ranging from 30 to 1500 μg/mL, for up to 3 days resulted in cell death in a dose- and time-dependent manner. Jurkat cell death was preceded by the appearance of multiple large vacuoles, which coincided with transcriptional up-regulation of the autophagy marker LC3 [30]. Distinct morphological changes including the appearance of large vacuoles, membrane disintegration and increased expression of autophagy protein LC3 transcripts indicated that fenugreek extract induced autophagy and autophagy-associated death of Jurkat cells [30].

Autophagy is a physiological response to stress and has been suggested to enable cells to adapt and survive and, hence, is considered a pro-survival mechanism [31]. Apoptosis is the best-described form of programmed cell death, but autophagy reportedly contributes to cell death as well. It has also been defined as an extremely conserved process of programmed cell death and its activation causes cell death [32]. Defective regulation of autophagy in cancers suggests that autophagy is a true tumor suppressor pathway [30].

It has been suggested that environmental and geographical differences may significantly influence the biologically active components of fenugreek extracts [24]. In our study, four different types of fenugreek were analyzed by proteomics fingerprinting. A set of eight protein spots from fenugreek seeds obtained from one particular region (A) were significantly different from those harvested from three different geographical locations (B, C & D). Our proteomics data, based on these eight protein spots, allowed accurate discrimination between regional fenugreek A and three other fenugreek samples B, C and D.

These proteins are of significant interest, and thus warrant further characterization to more fully explore their potential therapeutic use. We recognize the limited sample size of this preliminary study, however our results are of significant interest as they highlight the potential usefulness of a particular type of fenugreek as an anti-cancer drug. However, these exciting results obviously warrant additional study, including further drug characterization and development.

## Conclusions

In summary, to our knowledge, this is the first human case in which established malignant CNS cancer showed regression, then disappearing of the cancer lesion with daily use of fenugreek extract. Fenugreek may serve as a potential therapeutic in the treatment of lymphoid malignancy and other cancers. GC-MS analysis of fenugreek extract indicated the presence of several compounds with anticancer properties, including gingerol, cedrene, zingerone, vanillin and eugenol [30]. However whole crude extract has been shown to provide more selective potential than these individual compounds used in isolation [23]. Also it must be considered that the biologically active agents in fenugreek may vary based on geographical environments.

## Competing interest

The authors declare that they have no competing interest.

## Authors’ contribution

AA: Conception and design, review patient’s clinical data and results interpretation, /analysis and manuscript writing and editing. AF: designed /supervised in vitro experiments, assisted flow cytometry data analysis and interpretation, contributed to data analysis, interpretation and presentation. AA: Assisted Alkhodairy in carrying out in vitro experiments, apoptosis assays and flow cytometry experiments. A-MM: Assisted Alaiya in proteomics experiments, collection and assembly of data/interpretation. BE: Assisted Alaiya in proteomics experiments, collection and assembly of data/interpretation. SZ: Assisted Alaiya in proteomics experiments, collection and assembly of data/interpretation. AA: Designed, supervised, carried out, and analyzed proteomic data and interpretations, manuscript writing/editing. All authors read and approved the final manuscript.

## Pre-publication history

The pre-publication history for this paper can be accessed here:

http://www.biomedcentral.com/1472-6882/14/114/prepub

## Supplementary Material

Additional file 1: Figure S1 Brain MRI with contrast shows significant improvement of the new right frontoparietal intra cerebral lesion. Click here for file

Additional file 2: Figure S2 A, B: Brain MRI with contrast shows enhanced lesion. C, D: Brain MRI Flair sequence with new diffuse high signals over the right hemisphere. Click here for file

Additional file 3: Figure S3 Fenugreek exerted cytotoxicity effect on normal and cancer cells. The Cell-viability was measured by positive staining using propidium iodide. (LCL, Human Normal Lymphocytes, TCP are T-cell lymphoma, and FRO are the human Thyroid papillary carcinoma). Click here for file

Additional file 4: Figure S4 Flow cytometric graphs of T-cell lymphocytes (Normal cells) when treated with 300 μg/ml of Fenugreek, for 24, and 72 hours respectively. The very low number of cells migrated to the right- upper quadrant are the dead cells by apoptosis induction (late apoptosis) the right lower is the early apoptosis indicating that the apoptotic cell cytotoxicity of fenugreek is very low in normal T-cell lymphocytes cell line. Apoptosis and necrosis was measured using Annexin V apoptosis assay kit. (Molecular Probe). The graphical distribution of viable cells, necrotic and population of apoptotic cells was gated according to their staining signals to PI and Annexin V. The data was generated using a FACS caliber flow cytometry. Click here for file

Additional file 5: Figure S5 Apoptosis and cell viability in: Normal lymphocytes (LCL) (Blue)) showed no apoptosis, no cell killing, while the T-Cell lymphoma (Red column) and the Anaplastic Thyroid (FRO) (Green column); showed similar high cell cytotoxicity by apoptosis when incubated with fenugreek. Click here for file

Additional file 6: Table S1 Fenugreek cytotoxicity and Apoptotic effects on different cancer and normal cells. **Table S2.** Correlation analysis of pairs of 2-DE gels of fenugreek samples from four different regions A, B, C and D. Note the poor correlation between pairs of fenugreek from region A vs. B/C/D, compared with good correlation among pairs of D/C,B/D, C/B. Click here for file

Additional file 7: Figure S6 Global protein analysis showing expression levels of 8 protein spots that differs significantly between the fenugreek samples from region A and the rest three different regional fenugreek sample types using Student’s t- test. Click here for file

Additional file 8: Figure S7 AB: Gel segments of showing protein spots that are only present (A, upper panel) or absent (B, lower panel) in fenugreek samples from region A compared with the rest three other regional fenugreek samples B, C and D. Click here for file

## References

[B1] Losso JN Holliday DL Finley JW Martin RJ Rood JC Yu Y Greenway FL Fenugreek bread: a treatment for diabetes mellitus J Med Food 2009 12 5 1046 1049 19857068 10.1089/jmf.2008.0199

[B2] Sharma RD Raghuram TC Rao NS Effect of fenugreek seeds on blood glucose and serum lipids in type I diabetes Eur J Clin Nutr 1990 44 4 301 306 2194788

[B3] Uemura T Hirai S Mizoguchi N Goto T Lee JY Taketani K Nakano Y Shono J Hoshino S Tsuge N Narukami T Takahashi N Kawada T Diosgenin present in fenugreek improves glucose metabolism by promoting adipocyte differentiation and inhibiting inflammation in adipose tissues Mol Nutr Food Res 2010 54 11 1596 608 20540147 10.1002/mnfr.200900609

[B4] Pandian RS Anuradha CV Viswanathan P Gastroprotective effect of fenugreek seeds (Trigonella foenum graecum) on experimental gastric ulcer in rats J Ethnopharmacol 2002 81 3 393 397 12127242 10.1016/s0378-8741(02)00117-4

[B5] Khoja KK Shaf G Hasan TN Syed NA Al-Khalifa AS Al-Assaf AH Alshatwi AA Fenugreek, a naturally occurring edible spice, kills MCF-7 human breast cancer cells via an apoptotic pathway Asian Pac J Cancer Prev 2011 12 12 3299 3304 22471470

[B6] al-Ghamdi H Sabbah R Martin J Patay Z Primary T-cell lymphoma of the brain in children: a case report and literature review Pediatr Hematol Oncol 2000 17 4 341 343 10845234 10.1080/088800100276343

[B7] Ali L Azad Khan AK Hassan Z Mosihuzzaman M Nahar N Nasreen T Nur-e-Alam M Rokeya B Characterization of the hypoglycemic effects of Trigonella foenum graecum seed Planta Med 1995 61 4 358 360 7480183 10.1055/s-2006-958100

[B8] Navayath S Thiyagarajan D Fenugreek supplementation imparts erythrocyte resistance to cypermethrin induced oxidative changes in vivo J Complement Integr Med 2011 8 10.2202/1553-3840.1436 22754936

[B9] Al-Johar D Shinwari N Arif J Al-Sanea N Jabbar AA El-Sayed R Mashhour A Billedo G El-Doush I Al-Saleh I Role of Nigella sativa and a number of its antioxidant constituents towards azoxymethane-induced genotoxic effects and colon cancer in rats Phytother Res 2008 22 10 1311 1323 18570215 10.1002/ptr.2487

[B10] Alaiya AA Franzen B Hagman A Dysvik B Roblick UJ Becker S Moberger B Auer G Linder S Molecular classification of borderline ovarian tumors using hierarchical cluster analysis of protein expression profiles Int J Cancer 2002 98 6 895 899 11948469 10.1002/ijc.10288

[B11] Alaiya AA Franzen B Hagman A Silfversward C Moberger B Linder S Auer G Classification of human ovarian tumors using multivariate data analysis of polypeptide expression patterns Int J Cancer 2000 86 5 731 736 10797298 10.1002/(sici)1097-0215(20000601)86:5<731::aid-ijc20>3.0.co;2-a

[B12] Grant JW Isaacson PG Primary central nervous system lymphoma Brain Pathol 1992 2 2 97 109 1341961 10.1111/j.1750-3639.1992.tb00677.x

[B13] Bednar MM Salerni A Flanagan ME Pendlebury WW Primary central nervous system T-cell lymphoma. Case report J Neurosurg 1991 74 4 668 672 2002385 10.3171/jns.1991.74.4.0668

[B14] Knorr JR Ragland RL Stone BB Woda BA Gelber ND Cerebellar T-cell lymphoma: an unusual primary intracranial neoplasm Neuroradiology 1992 35 1 79 81 1289744 10.1007/BF00588285

[B15] Grant JW von Deimling A Primary T-cell lymphoma of the central nervous system Arch Pathol Lab Med 1990 114 1 24 27 2403777

[B16] Morgello S Maiese K Petito CK T-cell lymphoma in the CNS: clinical and pathologic features Neurology 1989 39 9 1190 1196 2788832 10.1212/wnl.39.9.1190

[B17] Shenkier TN1 Blay JY O’Neill BP Poortmans P Thiel E Jahnke K Abrey LE Neuwelt E Tsang R Batchelor T Harris N Ferreri AJ Ponzoni M O’Brien P Rubenstein J Connors JM Primary CNS lymphoma of T-cell origin: a descriptive analysis from the international primary CNS lymphoma collaborative group J Clin Oncol 2005 23 10 2233 2239 15800313 10.1200/JCO.2005.07.109

[B18] Latta S Myint ZW Jallad B Hamdi T Alhosaini MN Kumar DV Kheir F Primary central nervous system T-cell lymphoma in aids patients: case report and literature review Curr Oncol 2010 17 5 63 66 20975881 10.3747/co.v17i5.621 PMC2949374

[B19] Abrey LE Yahalom J DeAngelis LM Treatment for primary CNS lymphoma: the next step J Clin Oncol 2000 18 17 3144 3150 10963643 10.1200/JCO.2000.18.17.3144

[B20] Olaku O White JD Herbal therapy use by cancer patients: a literature review on case reports Eur J Cancer 2011 47 4 508 514 21185719 10.1016/j.ejca.2010.11.018 PMC3057114

[B21] Hibasami H Moteki H Ishikawa K Katsuzaki H Imai K Yoshioka K Ishii Y Komiya T Protodioscin isolated from fenugreek (Trigonella foenumgraecum L.) induces cell death and morphological change indicative of apoptosis in leukemic cell line H-60, but not in gastric cancer cell line KATO III Int J Mol Med 2003 11 1 23 26 12469212

[B22] Sur P Das M Gomes A Vedasiromoni JR Sahu NP Banerjee S Sharma RM Ganguly DK Trigonella foenum graecum (fenugreek) seed extract as an antineoplastic agent Phytother Res 2001 15 3 257 259 11351364 10.1002/ptr.718

[B23] Shabbeer S Sobolewski M Anchoori RK Kachhap S Hidalgo M Jimeno A Davidson N Carducci MA Khan SR Fenugreek: a naturally occurring edible spice as an anticancer agent Cancer Biol Ther 2009 8 3 272 278 19197146 10.4161/cbt.8.3.7443 PMC3095649

[B24] Raju J Patlolla JM Swamy MV Rao CV Diosgenin, a steroid saponin of Trigonella foenum graecum (Fenugreek), inhibits azoxymethane-induced aberrant crypt foci formation in F344 rats and induces apoptosis in HT-29 human colon cancer cells Cancer Epidemiol Biomarkers Prev 2004 13 8 1392 1398 15298963

[B25] Corbiere C Liagre B Bianchi A Bordji K Dauca M Netter P Beneytout JL Different contribution of apoptosis to the antiproliferative effects of diosgenin and other plant steroids, hecogenin and tigogenin, on human 1547 osteosarcoma cells Int J Oncol 2003 22 4 899 905 12632085

[B26] Liu MJ Wang Z Ju Y Wong RN Wu QY Diosgenin induces cell cycle arrest and apoptosis in human leukemia K562 cells with the disruption of Ca2+ homeostasis Cancer Chemother Pharmacol 2005 55 1 79 90 15372201 10.1007/s00280-004-0849-3

[B27] Srinivasan S Koduru S Kumar R Venguswamy G Kyprianou N Damodaran C Diosgenin targets Akt-mediated prosurvival signaling in human breast cancer cells Int J Cancer 2009 125 4 961 967 19384950 10.1002/ijc.24419

[B28] Li F Fernandez PP Rajendran P Hui KM Sethi G Diosgenin, a steroidal saponin, inhibits STAT3 signaling pathway leading to suppression of proliferation and chemosensitization of human hepatocellular carcinoma cells Cancer Lett 2010 292 2 197 207 20053498 10.1016/j.canlet.2009.12.003

[B29] Amin A Alkaabi A Al-Falasi S Daoud SA Chemopreventive activities of Trigonella foenum graecum (Fenugreek) against breast cancer Cell Biol Int 2005 29 8 687 694 15936223 10.1016/j.cellbi.2005.04.004

[B30] Al-Daghri NM Alokail MS Alkharfy KM Mohammed AK Abd-Alrahman SH Yakout SM Amer OE Krishnaswamy S Fenugreek extract as an inducer of cellular death via autophagy in human T lymphoma Jurkat cells BMC Complement Altern Med 2012 12 202 23110539 10.1186/1472-6882-12-202 PMC3520713

[B31] Chen N Debnath J Autophagy and tumorigenesis FEBS Lett 2010 584 7 1427 1435 20035753 10.1016/j.febslet.2009.12.034 PMC2843775

[B32] Vicencio JM1 Galluzzi L Tajeddine N Ortiz C Criollo A Tasdemir E Morselli E Ben Younes A Maiuri MC Lavandero S Kroemer G Senescence, apoptosis or autophagy? When a damaged cell must decide its path--a mini-review Gerontology 2008 54 2 92 99 18451641 10.1159/000129697

