# Supplementary figures and images for: The selective cytotoxic anti-cancer properties and proteomic analysis of Trigonella Foenum-Graecum
Source: BMC Complement Altern Med. 2014 Mar 29;14:114. doi: 10.1186/1472-6882-14-114 (PMC4021494; doi:10.1186/1472-6882-14-114)

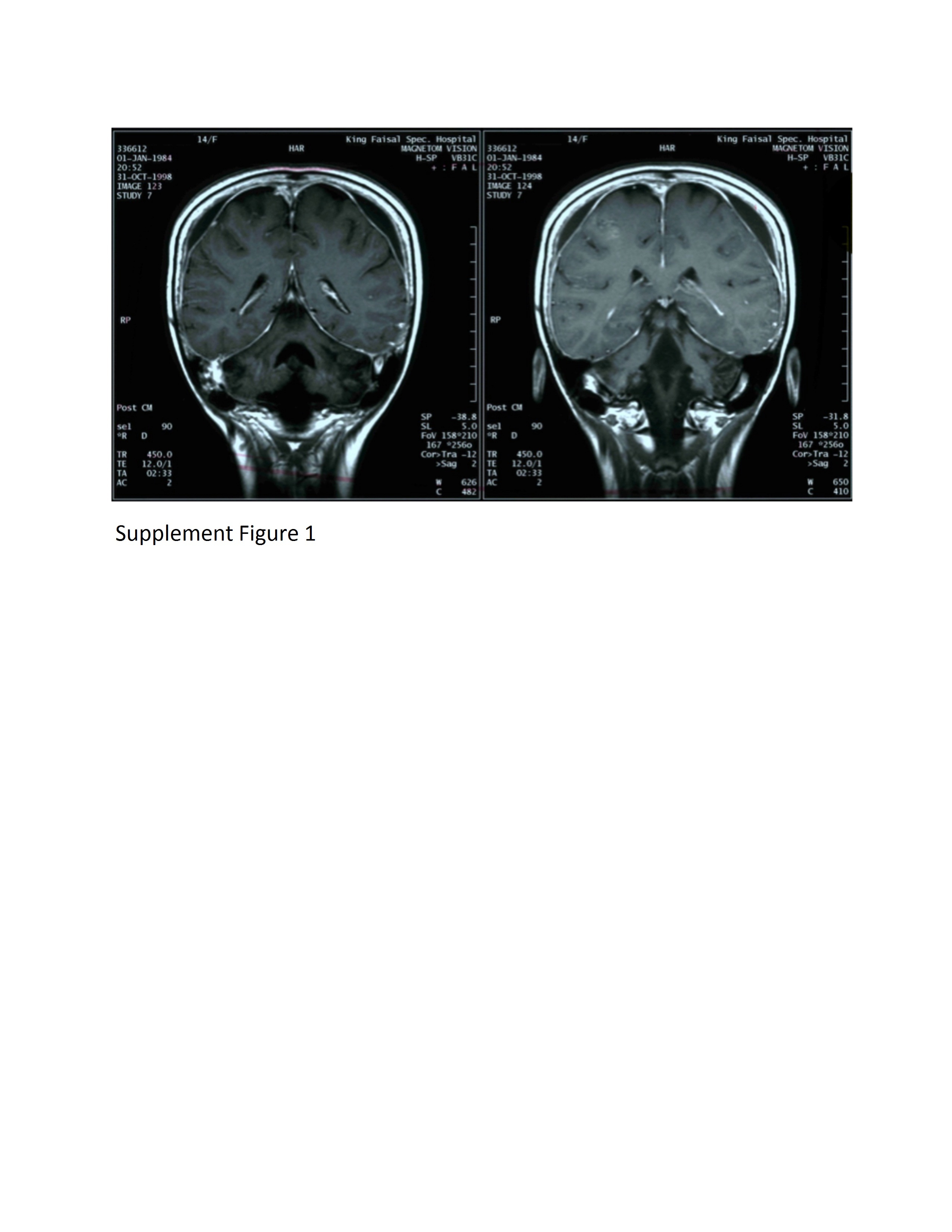

Supplement: Additional file 1: Figure S1 — Brain MRI with contrast shows significant improvement of the new right frontoparietal intra cerebral lesion. [file 1472-6882-14-114-S1.jpeg]

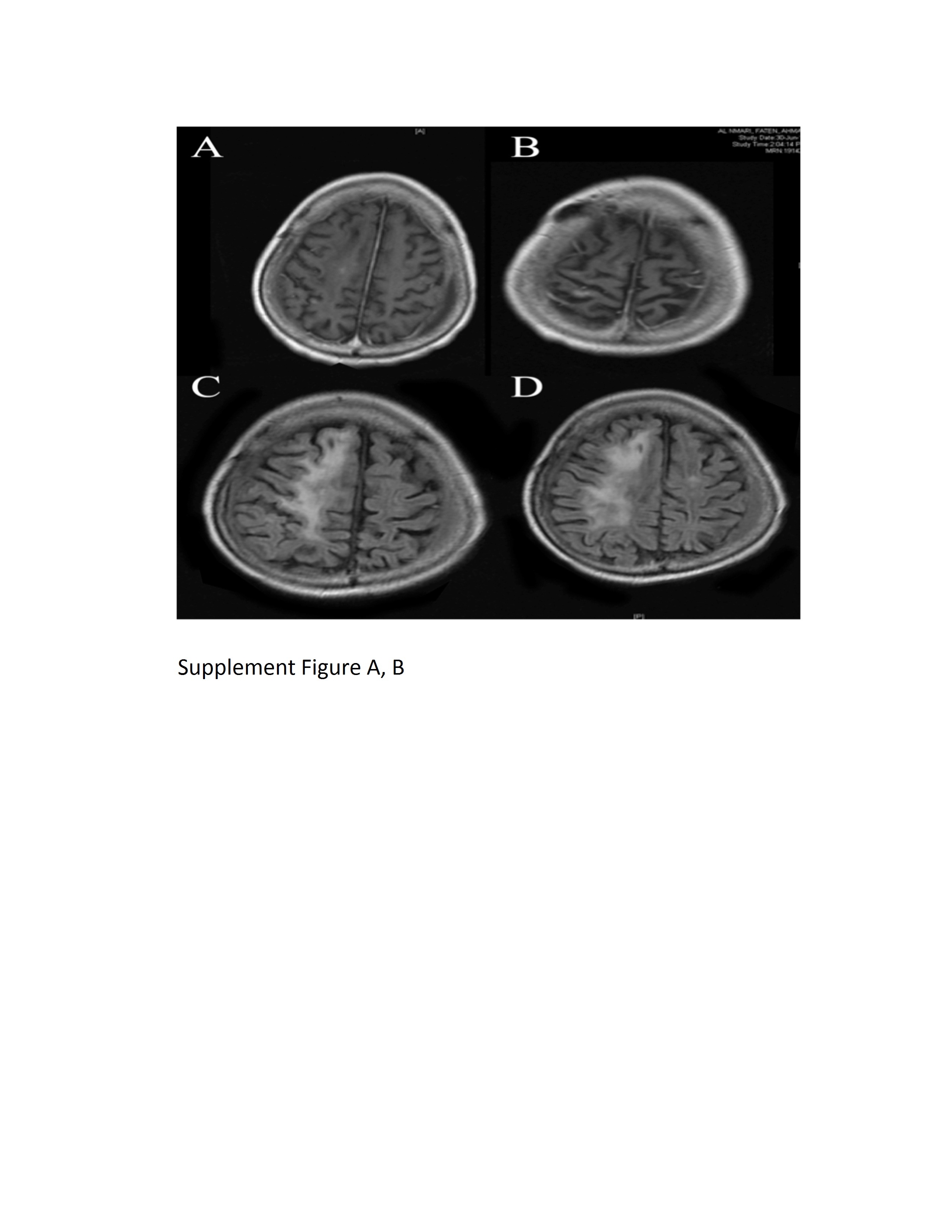

Supplement: Additional file 2: Figure S2 — A, B: Brain MRI with contrast shows enhanced lesion. C, D: Brain MRI Flair sequence with new diffuse high signals over the right hemisphere. [file 1472-6882-14-114-S2.jpeg]

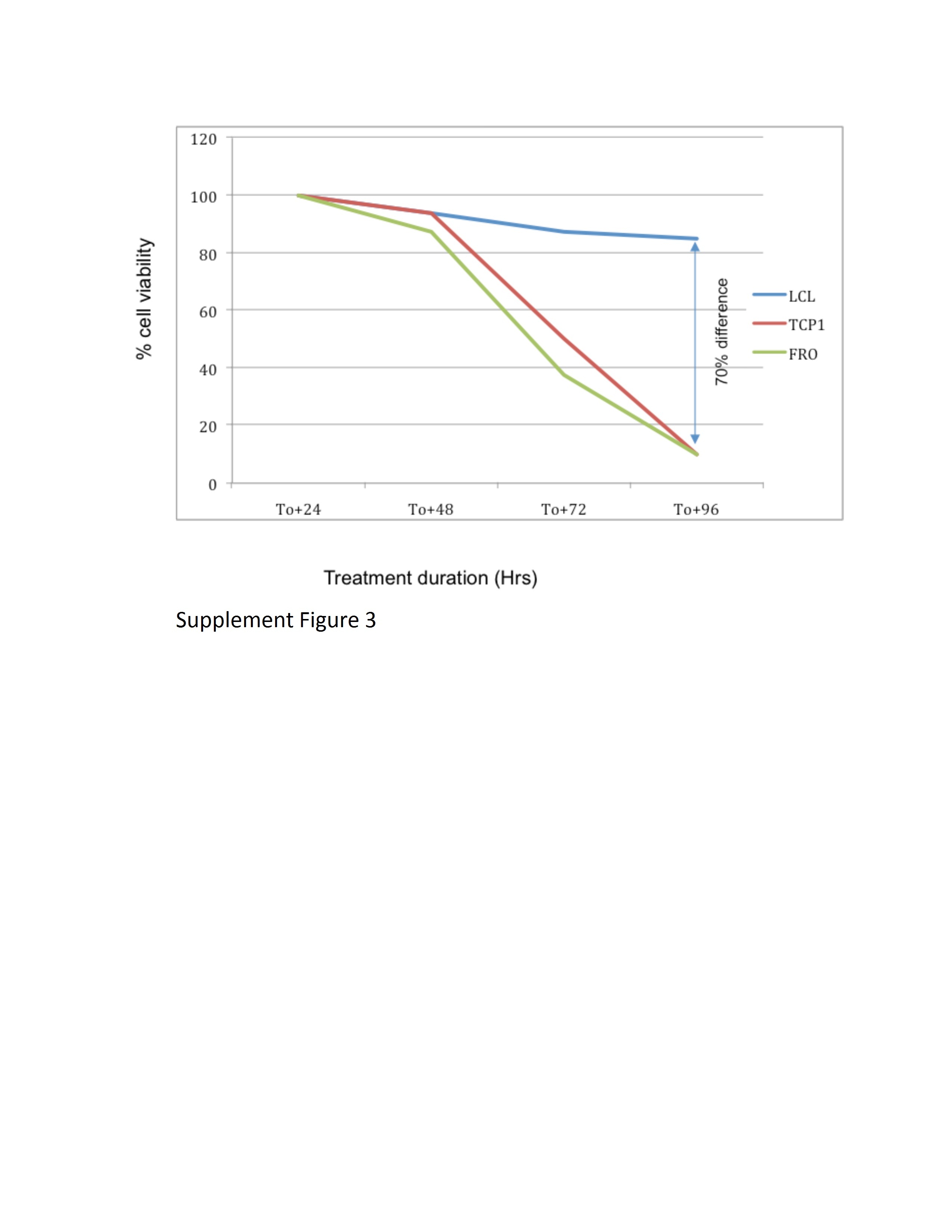

Supplement: Additional file 3: Figure S3 — Fenugreek exerted cytotoxicity effect on normal and cancer cells. The Cell-viability was measured by positive staining using propidium iodide. (LCL, Human Normal Lymphocytes, TCP are T-cell lymphoma, and FRO are the human Thyroid papillary carcinoma). [file 1472-6882-14-114-S3.jpeg]

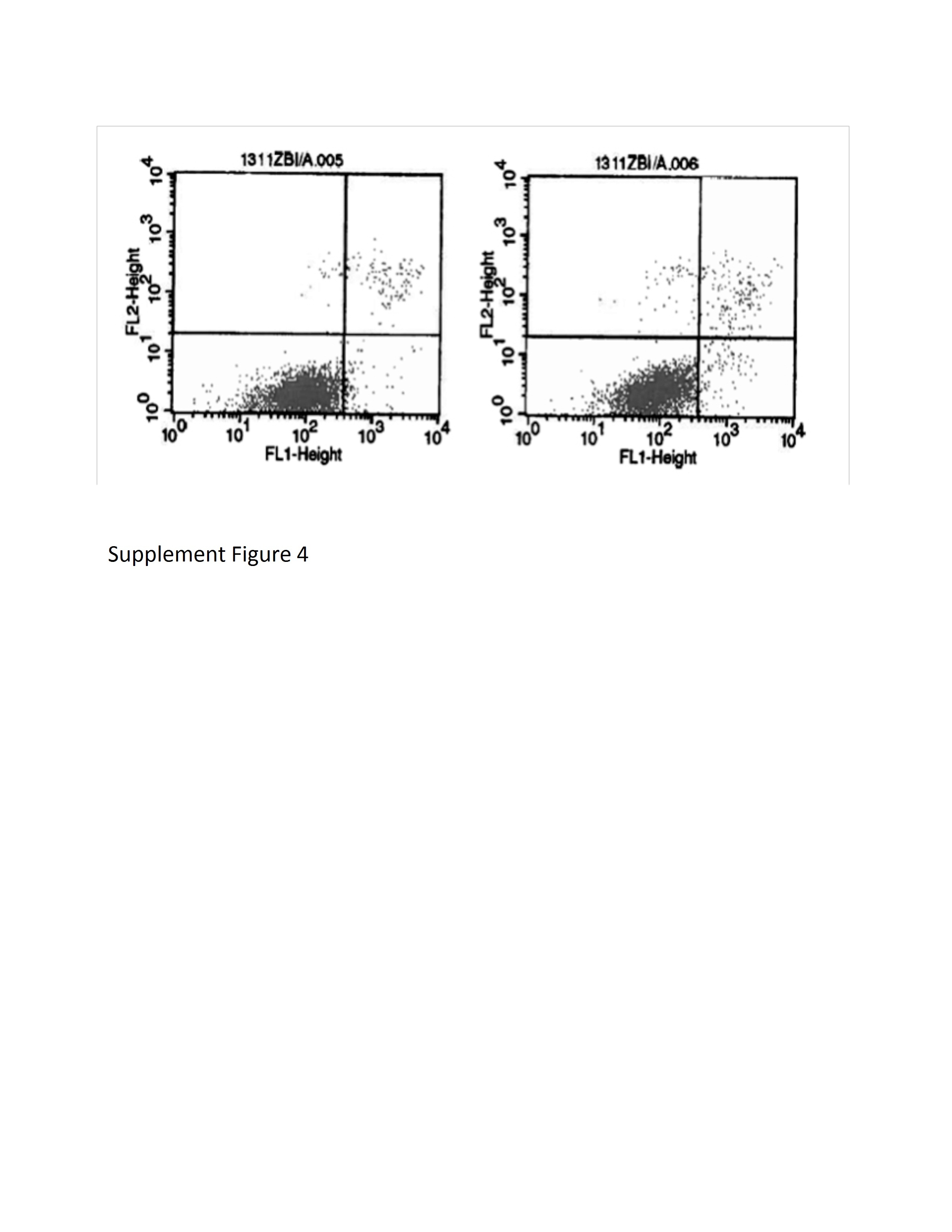

Supplement: Additional file 4: Figure S4 — Flow cytometric graphs of T-cell lymphocytes (Normal cells) when treated with 300 μg/ml of Fenugreek, for 24, and 72 hours respectively. The very low number of cells migrated to the right- upper quadrant are the dead cells by apoptosis induction (late apoptosis) the right lower is the early apoptosis indicating that the apoptotic cell cytotoxicity of fenugreek is very low in normal T-cell lymphocytes cell line. Apoptosis and necrosis was measured using Annexin V apoptosis assay kit. (Molecular Probe). The graphical distribution of viable cells, necrotic and population of apoptotic cells was gated according to their staining signals to PI and Annexin V. The data was generated using a FACS caliber flow cytometry. [file 1472-6882-14-114-S4.jpeg]

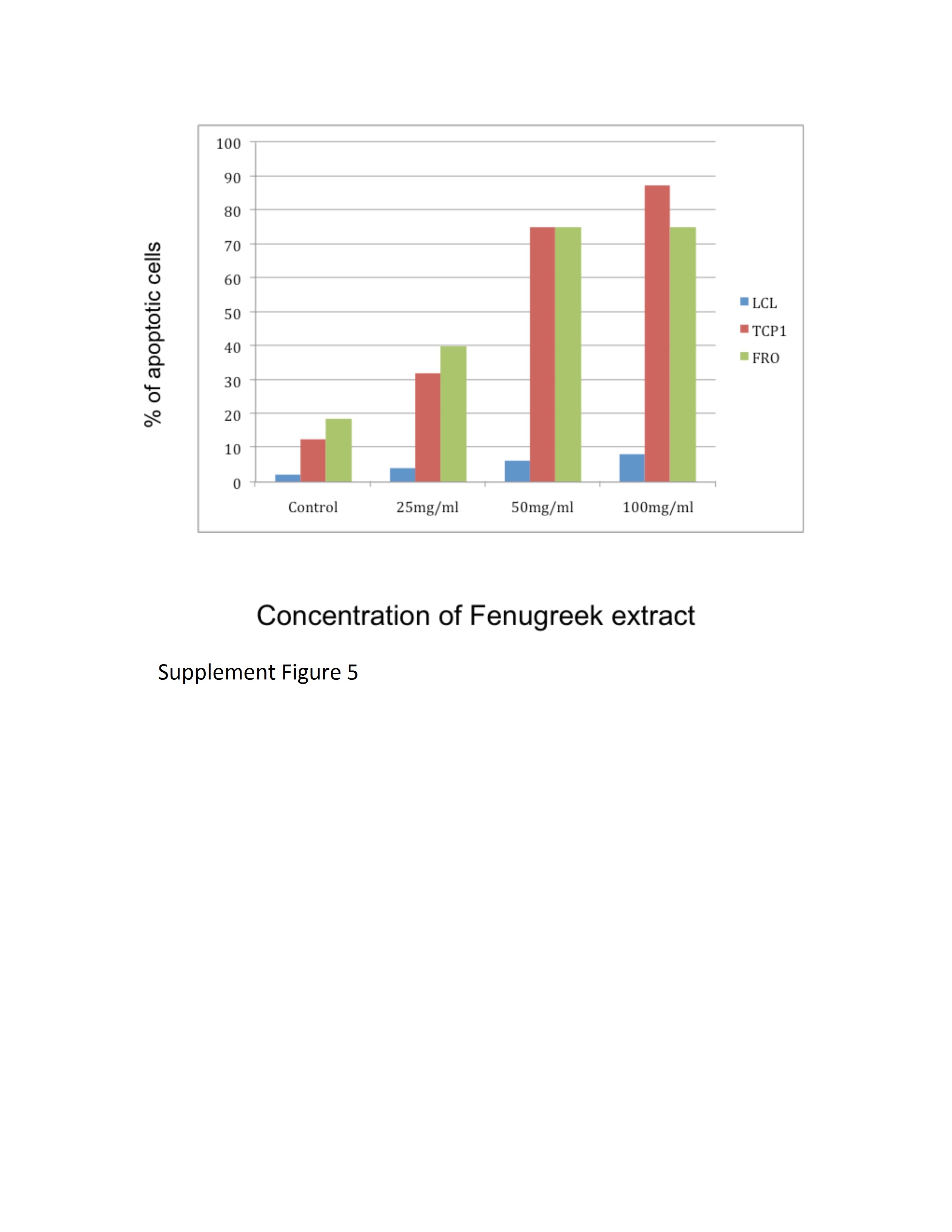

Supplement: Additional file 5: Figure S5 — Apoptosis and cell viability in: Normal lymphocytes (LCL) (Blue)) showed no apoptosis, no cell killing, while the T-Cell lymphoma (Red column) and the Anaplastic Thyroid (FRO) (Green column); showed similar high cell cytotoxicity by apoptosis when incubated with fenugreek. [file 1472-6882-14-114-S5.jpeg]

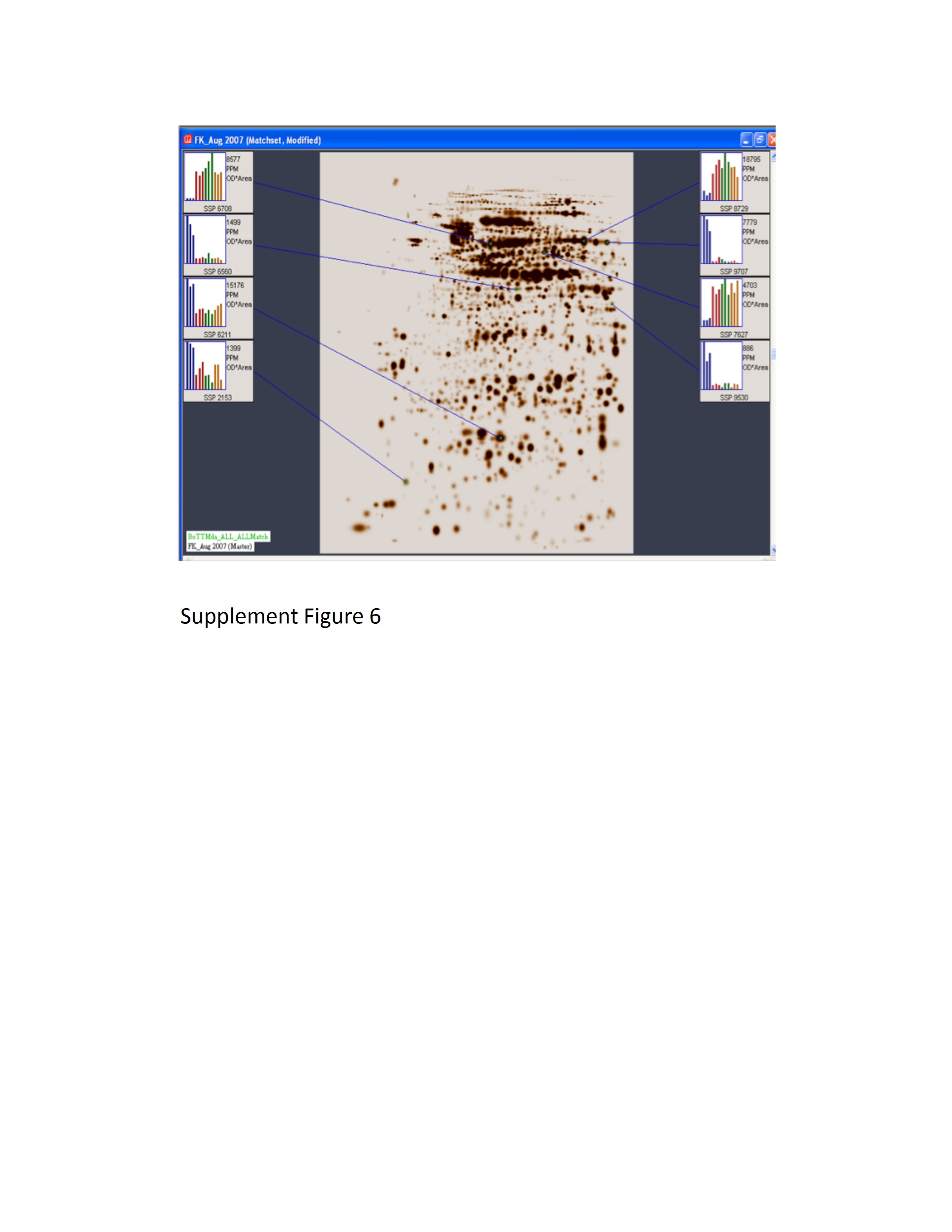

Supplement: Additional file 7: Figure S6 — Global protein analysis showing expression levels of 8 protein spots that differs significantly between the fenugreek samples from region A and the rest three different regional fenugreek sample types using Student’s t- test. [file 1472-6882-14-114-S7.jpeg]

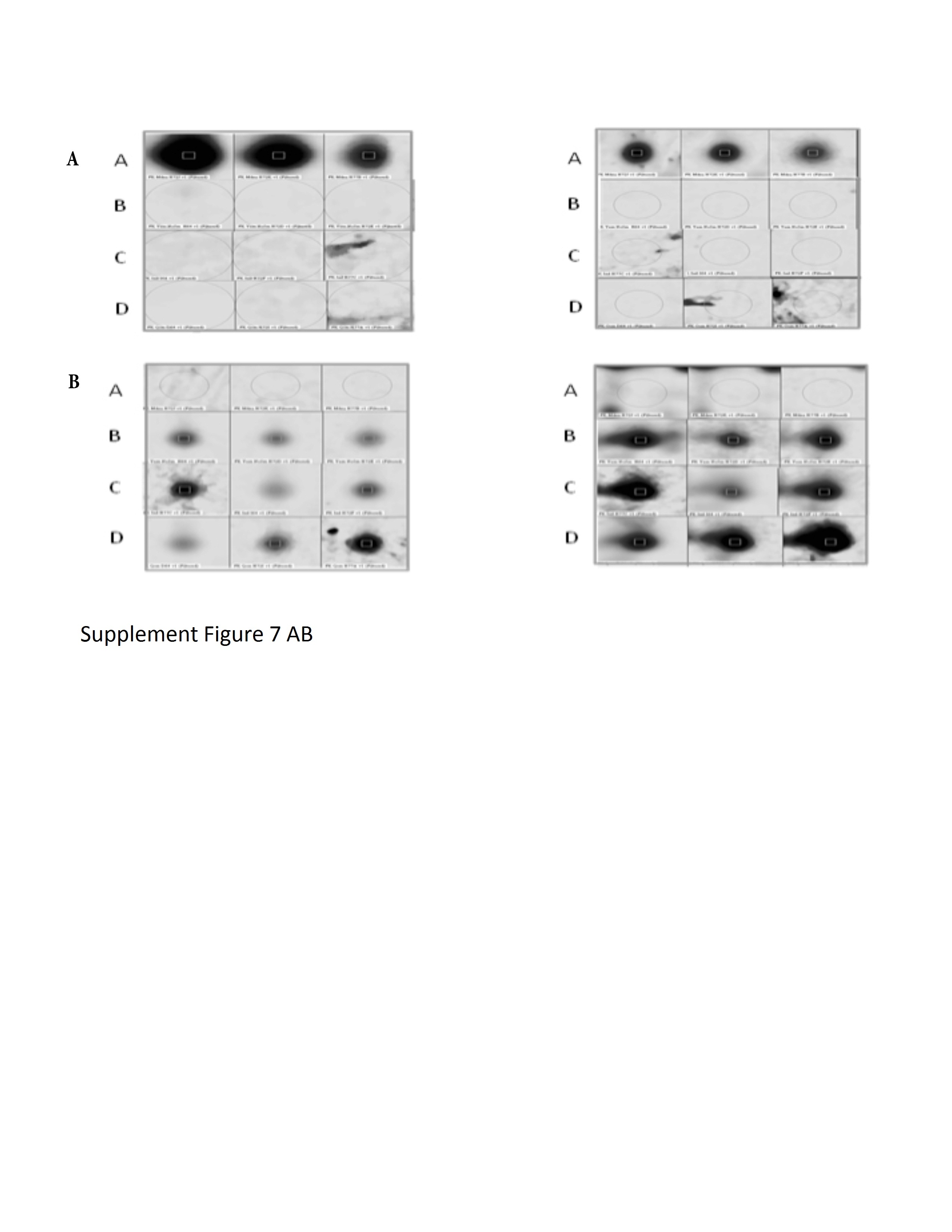

Supplement: Additional file 8: Figure S7 — AB: Gel segments of showing protein spots that are only present (A, upper panel) or absent (B, lower panel) in fenugreek samples from region A compared with the rest three other regional fenugreek samples B, C and D. [file 1472-6882-14-114-S8.jpeg]
