# Supplementary material for: The selective cytotoxic anti-cancer properties and proteomic analysis of Trigonella Foenum-Graecum
Source: BMC Complement Altern Med. 2014 Mar 29;14:114. doi: 10.1186/1472-6882-14-114 (PMC4021494; doi:10.1186/1472-6882-14-114)
Supplement: Additional file 6: Table S1 — Fenugreek cytotoxicity and Apoptotic effects on different cancer and normal cells. Table S2. Correlation analysis of pairs of 2-DE gels of fenugreek samples from four different regions A, B, C and D. Note the poor correlation between pairs of fenugreek from region A vs. B/C/D, compared with good correlation among pairs of D/C,B/D, C/B. [file 1472-6882-14-114-S6.doc]

| Cell Types | Cytotoxicity at 0 mg/ml (%) | Cytotoxcicity at 100 m g/ml (%) | Apoptosis (%)at 0 m g/ml (%) | Apoptosis (%) at100 mg/ml(%) |
| --- | --- | --- | --- | --- |
| LCL-Human Normal Lymphocytes | 2.5 | 8.5 | 4.0 | 18.2 |
| TCP -T-cell lymphoma | 12.5 | 87.5 | 13.5 | 72.6 |
| FRO -Human Thyroid papillary carcinoma | 18.5 | 75.0 | 11.4 | 61.3 |
| Glioblastoma | 19.0 | 31.4 | 18.7 | 43.3 |
| Memingioma | 0.0 | 20.0 | 7.31 | 19.86 |

Supplement Table1- Fenugreek cytotoxicity and Apoptotic effects on different cancer and normal cells

| Saple pairs | Correlation  coefficient (r) |
| --- | --- |
| Group correlation among pairs of A and B | 0.37 (n= 3 pairs) |
| Group correlation among pairs of A and D | 0.41 (n= 3 pairs) |
| Group correlation among pairs of A and C | 0.36 (n= 3 pairs) |
| Group correlation among pairs of B and D | 0.86 (n= 3 pairs) |
| Group correlation among pairs of B and C | 0.83 (n= 3 pairs) |
| Group correlation among pairs of D and C | 0.88 (n= 3 pairs) |

Supplement Table 2- Correlation analysis of pairs of 2-DE gels of fenugreek samples from four different regions A, B, C and D.Note the poor correlation between pairs of fenugreek from region A vs. B/C/D, compared with good correlation among pairs of D/C,B/D, C/B
